# Beyond *Anopheles gambiae* sensu lato: exploring the impact of non-dominant *Anopheles* species on malaria persistence in high-transmission endemic areas of Burkina Faso

**DOI:** 10.1186/s13071-025-07210-2

**Published:** 2026-01-05

**Authors:** Kelly L. Ngaffo, Aristide S. Hien, Dieudonné D. Soma, Samina Maiga, Emmanuel Sougue, Cheick Oumar W. Ouédraogo, Karama O. Delphine, Didier P. Alexandre Kaboré, Moussa Namountougou, Abdoulaye Diabaté, Etang D. Josiane, Roch K. Dabiré

**Affiliations:** 1 Institut de Recherche en Sciences de la Santé (IRSS), Direction Régionale de l’Ouest, BP 545, Bobo-Dioulasso, Burkina Faso; 2 Université Nazi Boni, Bobo-Dioulasso, Burkina Faso; 3 Laboratoire de Recherche sur le Paludisme, Institut de Recherche de Yaoundé, Organisation de Coordination pour la Lutte Contre les Endémies en Afrique Centrale, Yaoundé, Cameroon; 4 Université Cheikh Anta Diop de Dakar, Dakar, Senegal

**Keywords:** *Anopheles gambiae* sensu lato, Secondary malaria vectors, *Plasmodium falciparum*, Residual transmission, Burkina Faso

## Abstract

**Background:**

Despite widespread implementation of malaria control measures, including insecticide-treated nets (ITNs), indoor residual spraying (IRS), and seasonal malaria chemoprevention (SMC), malaria remains a major public health concern in Burkina Faso. The persistence of transmission is often attributed to increasing insecticide resistance in *Anopheles gambiae* sensu lato and drug resistance in *Plasmodium* spp. However, additional factors, such as climatic variability, ecological change, and shifts in vector species composition, may also play a role. This study investigated the geographic distribution of secondary malaria vectors and assessed their potential role in sustaining transmission at the national scale.

**Methods:**

Between 2023 and 2024, mosquito surveys were conducted across the three main ecological zones of Burkina Faso using human landing catches (HLC) and pyrethroid spray catches (PSC). Secondary vector species were identified morphologically. Molecular assays were used to detect *Plasmodium* infections and characterize blood-meal origins. Climatic data from national meteorological stations were analyzed to explore associations between environmental variables and species abundance.

**Results:**

A total of 1718 *Anopheles* mosquitoes (excluding *An. gambiae* s.l.) were collected, 688 in 2023 and 1030 in 2024. Five species were identified: *Anopheles nili*, *An. coustani*, *An. pharoensis*, *An. funestus*, and *An. rufipes*. Most specimens originated from the Sudan zone, with *An. nili* overwhelmingly dominant (87.5% of captures in 2023; 93% in 2024). Rainfall and, to a lesser extent, temperature were significantly associated with species abundance at several sites. Most mosquitoes were collected outdoors and showed strong anthropophilic tendencies. Molecular screening detected *Plasmodium falciparum* in all species except *An. funestus*. Infection was highest in Diébougou, with sporadic positive samples in *An. coustani* and *An. nili* across both years.

**Conclusions:**

Secondary vectors, particularly *An. nili* and *An. coustani*, appear to play an increasingly important role in malaria transmission in Burkina Faso. Their outdoor and sometimes opportunistic feeding behaviors highlight limitations of current indoor-focused interventions. These findings underscore the need to broaden surveillance and adapt vector control strategies to include secondary vector species in high-transmission settings.

**Graphical Abstract:**

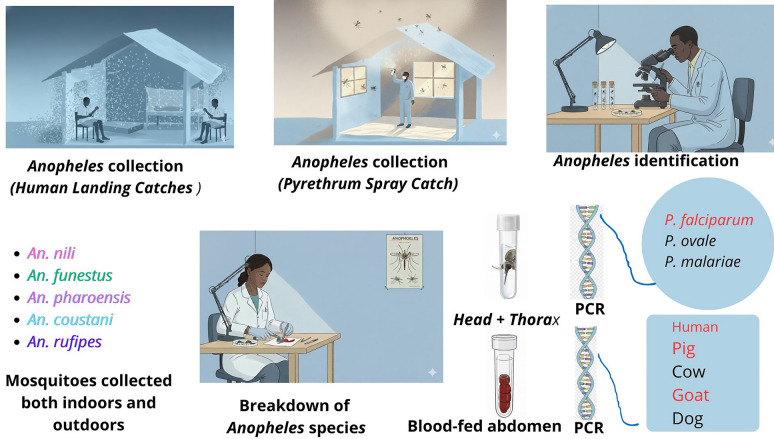

**Supplementary Information:**

The online version contains supplementary material available at 10.1186/s13071-025-07210-2.

## Background

Malaria remains one of the most significant public health challenges globally, with sub-Saharan Africa bearing the greatest burden. In 2023, an estimated 263 million malaria cases and approximately 597,000 deaths were recorded worldwide, highlighting the persistent difficulty of achieving sustained control [1]. Although major advances have been made through the deployment of malaria vaccines and vector-targeted interventions such as insecticide-treated nets (ITNs) and indoor residual spraying (IRS), progress has stalled in recent years, and several endemic regions are now experiencing a resurgence in cases [2–4]. This stagnation has intensified efforts to better understand the multifactorial drivers of persistent malaria transmission.

Several interconnected factors have been implicated in this plateau. These include the widespread emergence of insecticide resistance in *Anopheles* vectors and antimalarial drug resistance in *Plasmodium* parasites [5–7], as well as shifts in vector behavior, climatic variability, environmental change, and the recent incursion of the invasive vector *Anopheles stephensi* into multiple African countries [8–13]. Increasing ecological and biological heterogeneity, including species diversity, behavioral plasticity, and varying ecological adaptations, further complicates current control strategies, which primarily target a narrow group of dominant vector species [9, 14]. While *Anopheles gambiae* s.l., *An. funestus*, and a few others remain the most important malaria vectors in sub-Saharan Africa [15, 16], growing evidence highlights the epidemiological relevance of secondary vectors such as *An. pharoensis*, *An. coustani*, and *An. squamosus*, particularly in settings where primary vector populations have been reduced by control interventions [17–21].

Burkina Faso harbors a complex assemblage of *Anopheles* species with marked ecological and geographic heterogeneity in vector composition. Although *An. gambiae* s.l. is widespread and remains the principal target of national vector control efforts [22], other species, including *Anopheles funestus*, *An. nili*, *An. pharoensis*, *An. coustani*, and *An. squamosus*, are also present and may contribute to transmission [23–25]. These species exhibit diverse ecological preferences and behavioral traits that may reduce their susceptibility to ITNs and IRS, such as outdoor biting, exophilic resting behavior, and zoophagy [14, 26]. Despite their potential epidemiological importance, the contribution of secondary vectors to residual malaria transmission remains insufficiently studied in Burkina Faso, especially in the context of environmental change, widespread insecticide resistance, and shifts in vector population dynamics.

Addressing these knowledge gaps is crucial for optimizing malaria control strategies and improving surveillance systems. Therefore, this study aimed to characterize the spatial distribution, species composition, biting behavior, feeding patterns, and *Plasmodium* infection rates of secondary malaria vectors across diverse ecological zones of Burkina Faso. The findings provide critical insights into the potential role of these species in sustaining malaria transmission, particularly in high-transmission regions where primary vector control tools may have reduced effectiveness.

## Methods

### Study sites

**Fig. 1:**
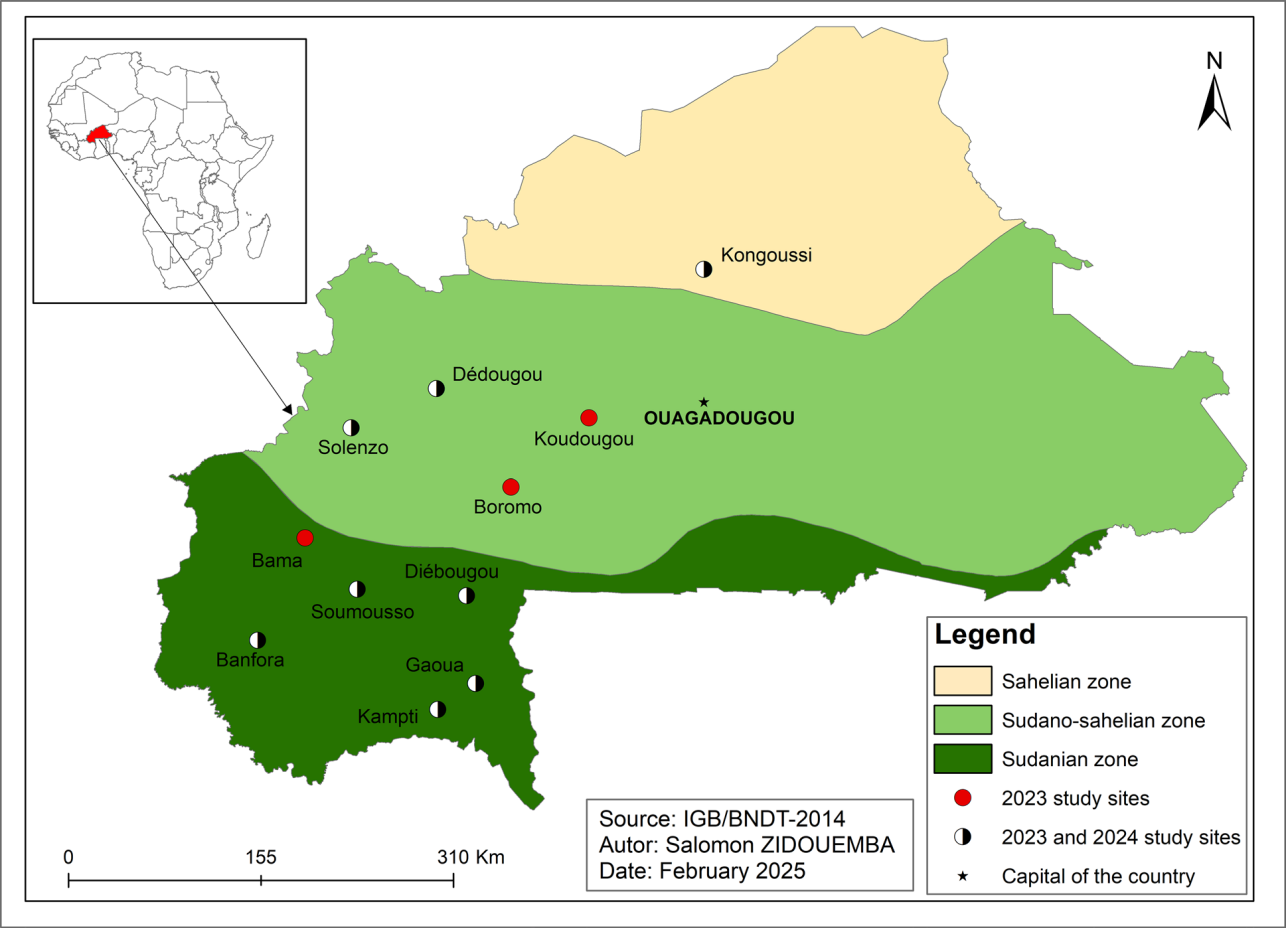
Study sites representing mosquito collection locations across the three ecological zones of Burkina Faso in 2023 and 2024. Black-and-white points indicate sites sampled in both 2023 and 2024, while red points indicate sites sampled only in 2023

Mosquito collections were conducted at 11 sites distributed across the three major eco-climatic zones of Burkina Faso: Sahelian, Sudano-Sahelian, and Sudanian (Fig. 1). Eight sites were originally selected for sampling in both 2023 and 2024; three additional sites (Bama, Boromo, and Koudougou) were included during the 2023 sampling period to increase geographic coverage.

The Sahelian zone, represented by Kongoussi, is characterized by an extended dry season lasting 8–9 months and annual rainfall < 600 mm. The Sudano-Sahelian zone includes Dédougou, Solenzo, Boromo, and Koudougou, where the dry season lasts 5–8 months and annual rainfall ranges between 600 and 900 mm. The Sudanian zone comprising Banfora, Kampti, Gaoua, Diébougou, Soumousso, and Bama is the most humid, with an approximately 6-month rainy season and annual precipitation between 1000 and 1200 mm. This southern region is considered a high-transmission area, where malaria transmission typically begins in May and persists throughout the year.

### Mosquito collection

Mosquitoes were sampled monthly from June to December in both 2023 and 2024 using human landing catches (HLC) and pyrethroid spray catches (PSC).

#### Human landing catches (HLC)

At each site, four houses were selected to ensure broad spatial representation. For 2 consecutive nights per month, indoor and outdoor collection points were established at each house. Collections were conducted from 6:00 p.m. to 8:00 p.m. using 5-ml hemolysis tubes to capture mosquitoes landing on exposed collectors’ legs before biting. All collectors were adult males who rotated positions to minimize collector bias. Field supervisors ensured vigilance throughout the night to reduce risk of mosquito bites.

Mosquitoes were stored separately by hour and by indoor/outdoor location. HLC is considered the gold standard for measuring human-vector contact and estimating transmission intensity, and it captures a broader diversity of mosquito species than most alternative sampling methods [27, 28].

#### Pyrethroid spray catches (PSC)

PSC was performed once per month in 20 randomly selected houses at each site to sample indoor-resting mosquitoes and to obtain freshly fed females for blood meal analysis. Early each morning, rooms were prepared by covering floors with white sheets and sealing openings. A non-residual pyrethroid was sprayed, and the room remained closed for 15 min. Knocked-down mosquitoes were collected using forceps and transferred to petri dishes for identification [29].

### Morphological identification

All *Anopheles* mosquitoes were identified morphologically using the Coetzee (2020) identification keys [30]. Specimens were classified according to abdominal physiological stage (unfed, fed, semi-gravid, gravid). The head-thorax of unfed females was retained for molecular analyses; head-thoraces from fed and gravid females were later included as needed. All samples were stored at −20 °C until processing.

### Molecular analysis

#### DNA extraction

Genomic DNA was extracted from the head-thorax portion of each mosquito using the 2% cetyltrimethylammonium bromide (CTAB) method [31]. Heads and thoraxes were placed individually in 1.5-ml tubes. Abdomen samples used for blood-meal analysis were placed in separate tubes containing 200 µl of 2% CTAB buffer and a sterile bead. Tissue homogenization was performed using a QIAGEN TissueLyser II. After extraction and precipitation, DNA pellets were resuspended in 20 µl of nuclease-free water.

#### *Plasmodium* spp. detection

A single-round multiplex PCR assay targeting species-specific small-subunit ribosomal RNA (SSU rRNA) sequences was used to detect *Plasmodium falciparum*, *P. ovale*, and *P. malariae* following Padley et al. [32]. Primers included:

- *Plasmodium* genus: GTA TCT GAT CGT CTT CAC TCC C
- *Plasmodium falciparum*: AAC AGA CGG GTA GTC ATG ATT GAG
- *Plasmodium ovale*: CTG TTC TTT GCA TTC CTT ATG C
- *Plasmodium malariae*: CGT TAA GAT AAA CGC CAA GCG

Reactions (15 µl) contained 7.5 µl of QIAGEN 2× Multiplex PCR Master Mix, 0.6 µl genus-specific primer (10 mM), 0.3 µl of each species-specific primer (10 mM), 3 µl water, and 3 µl genomic DNA. PCR conditions were: 95 °C for 15 min; 35 cycles of 95 °C for 30 s, 60 °C for 90 s, 72 °C for 90 s; final extension at 72 °C for 10 min.

#### Origin of blood meal

Abdomens of blood-fed secondary vectors were analyzed to determine host feeding preferences. Domestic and livestock animals vary across ecological zones but commonly include humans, cattle, goats, sheep, pigs, donkeys, dogs, and poultry. Blood-meal origin was determined using two multiplex PCRs targeting the cytochrome-b gene of common vertebrate hosts following Kent and Norris [33] (primer list in Supplementary Table 1). Each 20-µl reaction contained 5× FIREPol^®^ Master Mix. PCR cycling included: 95 °C for 5 min; 35 cycles of 95 °C for 30 s, 60 °C for 45 s, 72 °C for 60 s; final extension at 72 °C for 5 min.

### Data analysis

Data were analyzed using Microsoft Excel 2016 and R version 4.2.1. Vector abundance, species proportions, human biting rates (HBR: bites/person/night), and entomological inoculation rates (EIR: infective bites/person/night) were estimated. Chi-square tests were used to compare species proportions between years. To assess the influence of climatic factors (temperature and rainfall) on mosquito abundance, both bivariate and multivariate analyses were conducted. For each species and site, generalized linear models (GLMs) with a negative binomial distribution were fitted to account for overdispersion.

Statistical thresholds:

- *P* < 0.05: significant
- *P* < 0.005: moderately strong effect
- *P* < 0.0005: highly significant

## Results

### Composition and distribution of secondary vectors across eco-climatic zones

**Fig. 2:**
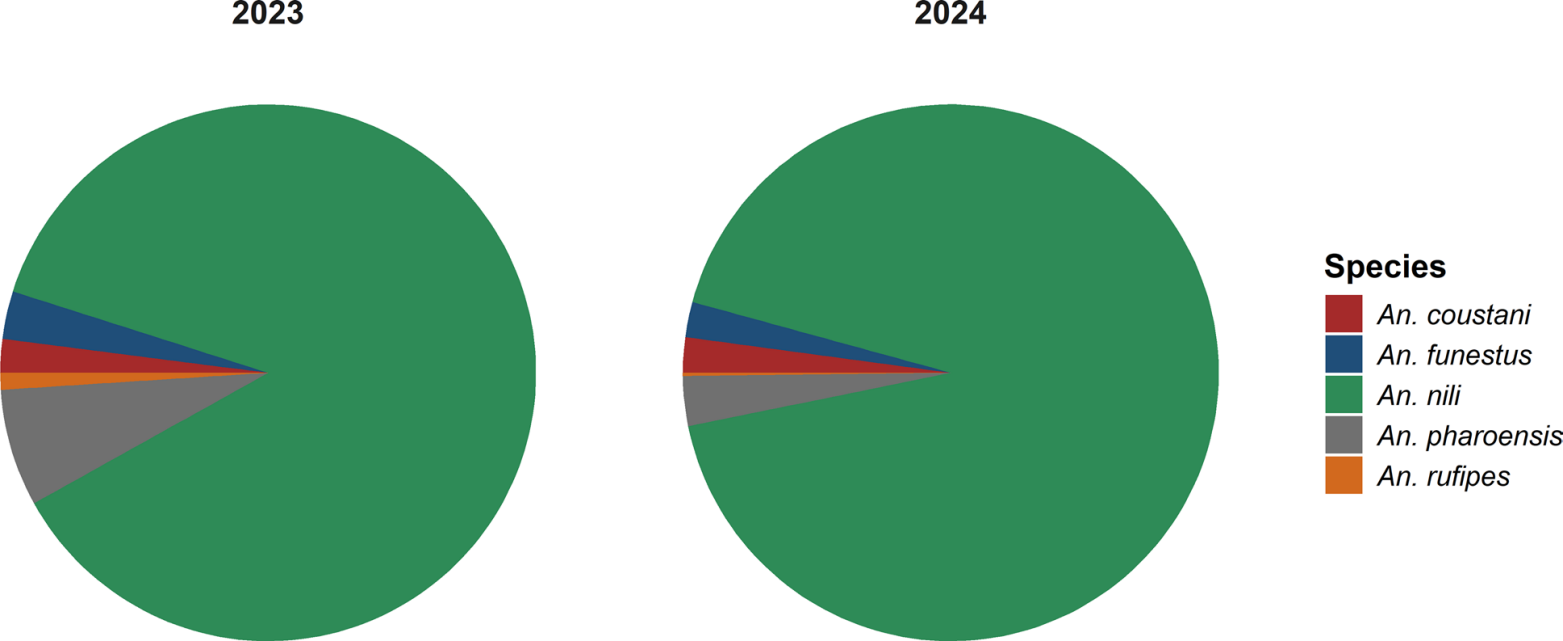
Composition and abundance of secondary vectors in mosquitoes collected across all study sites in 2023 and 2024

A total of 1718 *Anopheles* mosquitoes belonging to species other than *An. gambiae* s.l. were collected between June and December of 2023 and 2024. Human landing catches accounted for 1692 specimens, while 26 were collected via PSC (Fig. 2). Of these, 688 mosquitoes were collected in 2023 and 1030 in 2024.

**Fig. 3:**
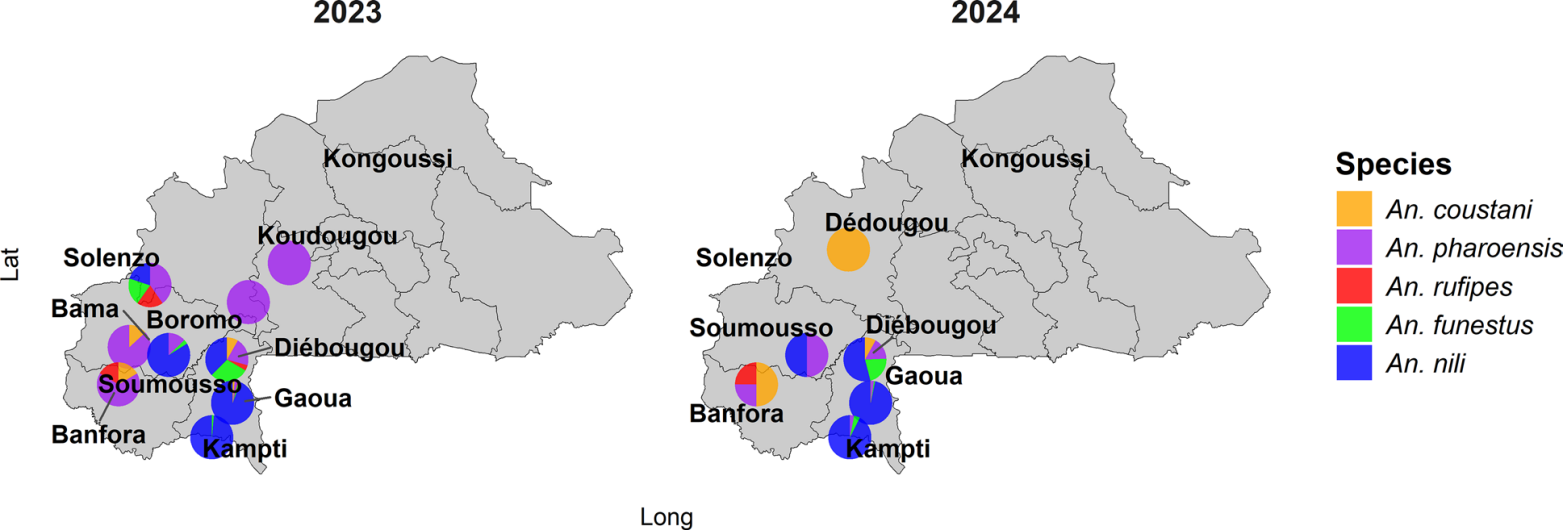
Distribution of secondary vector species across the climate areas of Burkina Faso in 2023 and 2024: Sites without pie charts are locations where secondary vectors were not found. Lat: latitude; Long: longitude

Five secondary vector species were identified: *Anopheles nili*, *An. coustani*, *An. pharoensis*, *An. funestus*, and *An. rufipes*. *Anopheles nili* was the most abundant species in both years, representing 87.5% (*n* = 602) of collections in 2023 and 93.0% (*n* = 958) in 2024 (combined HLC and PSC). In contrast, *An. rufipes* was the least represented, with only six individuals (0.87%) in 2023 and one (0.76%) in 2024 (Fig. 3). Species composition differed significantly between the 2 years (*χ*^2^ = 25.553, *df* = 6, *P* < 0.001).

No secondary *Anopheles* species were detected in the Sahelian zone. The Sudanian zone exhibited the highest species richness and abundance, with all five species present. In 2023, *An. pharoensis* was dominant in the Sudano-Sahelian zone, whereas in 2024, *An. coustani* was the only species detected there. Gaoua recorded the highest mosquito density, dominated by *An. nili*. Diébougou was the only site where all five species were recorded throughout the study period.

### Site-specific effects of climate on secondary vector species composition

**Fig. 4:**
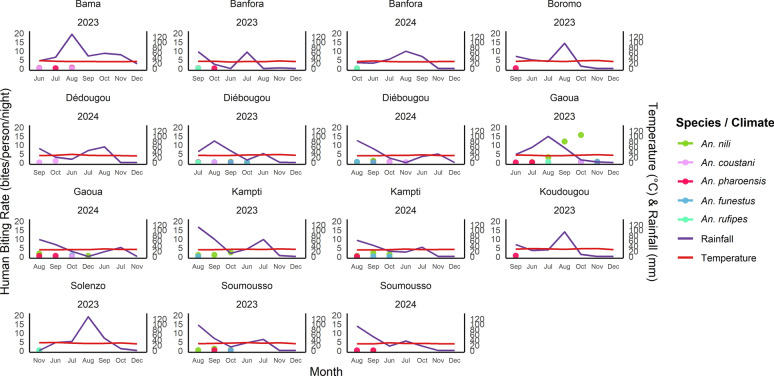
Number of *Anopheles* bites per person per night in relation to monthly temperature and precipitation levels across the study sites in 2023 and 2024

Monthly species distributions varied across sites, species, and years (Fig. 4). Mosquito abundance peaked from August to October, corresponding to the late rainy season, whereas temperature remained relatively stable across months and sites. *Anopheles pharoensis* was the most consistently detected species, occurring from June to September and occasionally into October, particularly in sites with rainfall > 30 mm. Other species (*An. nili*, *An. coustani*, *An. funestus*, *An. rufipes*) showed irregular, site-specific patterns. *Anopheles nili* and *An. coustani* were most frequently observed in October.

Significant positive correlations between rainfall and species abundance were found for *An. coustani* in Gaoua, *An. nili* in Diébougou and Kampti, and *An. funestus* in Diébougou (Table 1). A significant association with temperature was only detected for *An. nili* in Diébougou (*β* = −1.10; *P* = 0.025). In multivariate models, rainfall remained significantly associated with *An. coustani* abundance in Gaoua (*β* = 0.05; *P* < 0.0001). Conversely, *An. funestus* abundance in Diébougou showed a slight but significant negative association with rainfall (*β* = −0.03; *P* < 0.001). For *An. nili*, neither temperature nor rainfall remained significant in multivariate analysis.

**Table 1:** Analysis of correlation between rainfall and abundance of *Anopheles*

Species	Site	Rainfall coefficient (*β*)	Std. error	*P* value	Interpretation
*An. funestus*	Diébougou	0.0416	0.0102	< 0.0001	Strong positive effect of rainfall
*An. nili*	Kampti	0.0077	0.0025	0.0022	Moderate positive effect of rainfall
*An. nili*	Diébougou	0.0506	0.0183	0.0057	Significant positive effect
*An. coustani*	Gaoua	0.0513	0.0128	< 0.0001	Strong positive effect of rainfall

### Biting patterns of secondary malaria vectors

#### Human biting rates (HBR)

**Fig. 5:**
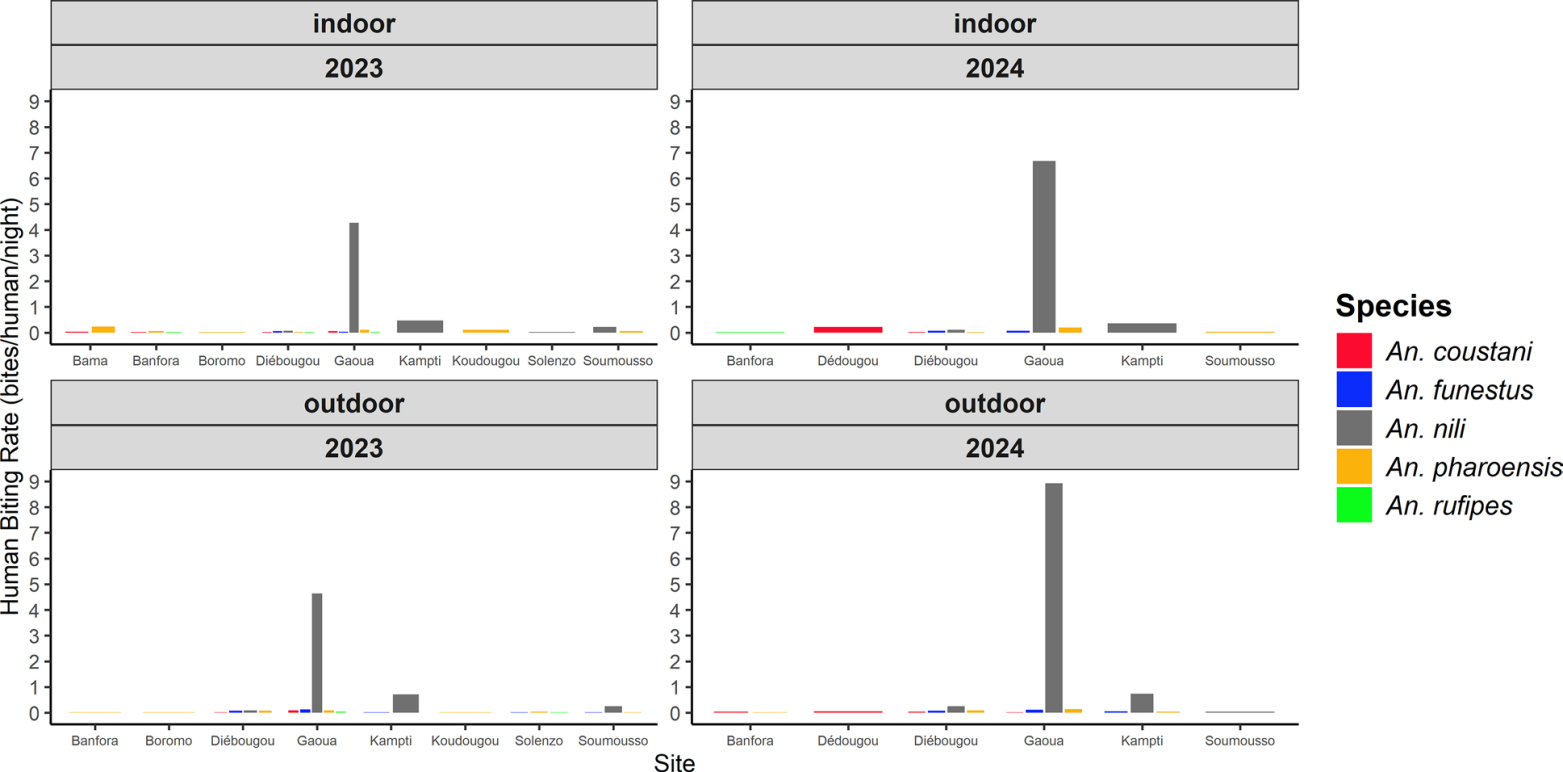
Indoor and outdoor human-biting rate of *Anopheles* mosquitoes in 2023 and 2024. The numbers within the graphs indicate the number of bites per person per night

Human biting rates for the five secondary vector species are presented in Fig. 5 for indoor and outdoor settings across sites and years. HBRs varied broadly by species, site, and year. The lowest HBRs were 0.02 bites per person per night (b/p/n), observed for several species across multiple sites in both years. The highest HBRs were recorded for *An. nili* in Gaoua, which exhibited elevated biting both indoors and outdoors in 2023 corresponding to 5.69 b/p/n (indoors) and 6.19 b/p/n (outdoors). In 2024, HBRs were 8.90 b/p/n (indoors) and 11.90 b/p/n (outdoors). Most secondary vectors were collected outdoors, but *An. coustani*, *An. pharoensis*, and *An. rufipes* bite both indoors and outdoors, with site-specific variation. *Anopheles nili* and *An. funestus* were predominantly exophagic.

#### Hourly biting patterns

**Fig. 6:**
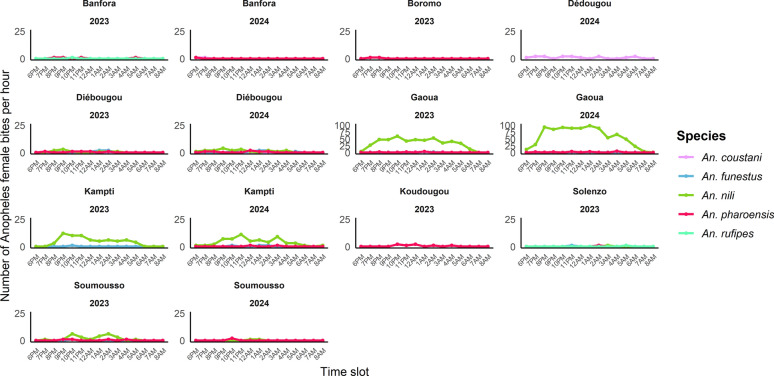
Total number of *Anopheles* mosquitoes (indoor and outdoor) biting at each collection hour across all study sites in 2023 and 2024

Secondary vector species exhibited distinct hourly biting profiles across sites and years (Fig. 6). Activity generally spanned the full night: In 2023, biting started around 6:00 p.m. and ended by 06:00 a.m. In 2024, activity extended until 8:00 a.m. The highest peak was recorded for *An. nili* in Gaoua in 2023 between 8:00 p.m. and 9:00 p.m. and in 2024 between 9:00 p.m. and 10:00 p.m.

Species-specific observations included:

- *Anopheles rufipes*: limited activity (only 10:00 p.m. and 5:00 a.m.).
- *Anopheles pharoensis*: broad activity window (6:00 p.m. to 4:00 a.m.).
- *Anopheles coustani*: active from 6:00 p.m. to 6:00 a.m., occasionally showing early evening biting.
- *Anopheles funestus*: activity from 7:00 p.m. to 6:00 a.m.
- *Anopheles nili*: continuous biting throughout the night with strong early evening peaks.

Among all species, *An. nili* was the only one showing a clear and consistent biting pattern.

### Host-feeding patterns of secondary vectors

A total of 334 blood-fed mosquitoes were analyzed: 299 *An. nili*, 11 *An. funestus*, 14 *An. pharoensis*, 7 *An. coustani*, and 3 *An. rufipes* (Table 2). Strong interspecific and spatial variations in host-feeding behavior were observed. *Anopheles nili* showed a pronounced anthropophilic tendency particularly in Gaoua, where 66.4% fed exclusively on humans and 10.9% contained mixed human-animal blood meals.

**Table 2:** *Anopheles* blood meal sources in our study sites

Site	Species	Human		Human/goat		Animal				Total
		*n*	%	*n*	%	Goat	Pig	Other	%	
Bama	*An. coustani*	0	0	0	0	0	0	1	100	1
	*An. pharoensi*	1	16.66	0	0	0	0	5	83.33	6
Banfora	*An. pharoensi*	1	100	0	0	0	0	0	0	1
	*An. rufipes*	1	1	0	0	0	0	0	0	1
Dédougou	*An. coustani*	3	100	0	0	0	0	0	0	3
Diebougou	*An. funestus*	2	50	1	25	0	0	1	25	4
	*An. nili*	1	25	1	25	0	1	1	50	4
	*An. pharoensi*	1	50	1	50	0	0	0	0	2
	*An. rufipes*	0	0	0	0	0	0	1	100	1
Gaoua	*An. coustani*	1	25	0	0	0	0	2	75	3
	*An. funestus*	3	50	1	16.66	0	0	2	33.33	6
	*An. nili*	164	66.39	27	10.93	5	0	51	22.67	247
	*An. pharoensi*	2	50	0	0	1	0	1	50	4
Kampti	*An. funestus*	0	0	1	100	0	0	0	0	1
	*An. nili*	21	53.84	3	7.69	0	0	15	38.46	39
Solenzo	*An. rufipes*	0	0	0	0	1	0	0	100	1
Soumousso	*An. nili*	6	66.6	2	22.22	0	0	1	11.11	9
	*An. pharoensi*	1	100	0	0	0	0	0	0	1

*An*: *Anopheles*; *n*: number of blood-fed *Anopheles* females; *Other*: blood meal sources undetermined; Human/goat: fed on both human and goat

Other species were predominantly zoophilic or opportunistic:

- *Anopheles rufipes* (Solenzo, Diébougou): 100% animal blood
- *Anopheles coustani* (Bama, Gaoua): 75–100% animal blood

Mixed blood meals occurred in several species, including *An. funestus*, *An. nili*, and *An. pharoensis*, suggesting opportunistic feeding and possible bridge-vector roles. Spatially, Gaoua showed the highest frequency of human-fed mosquitoes, while Bama, Banfora, and Solenzo had the lowest anthropophagic rates.

### Temporal feeding dynamics

**Fig. 7:**
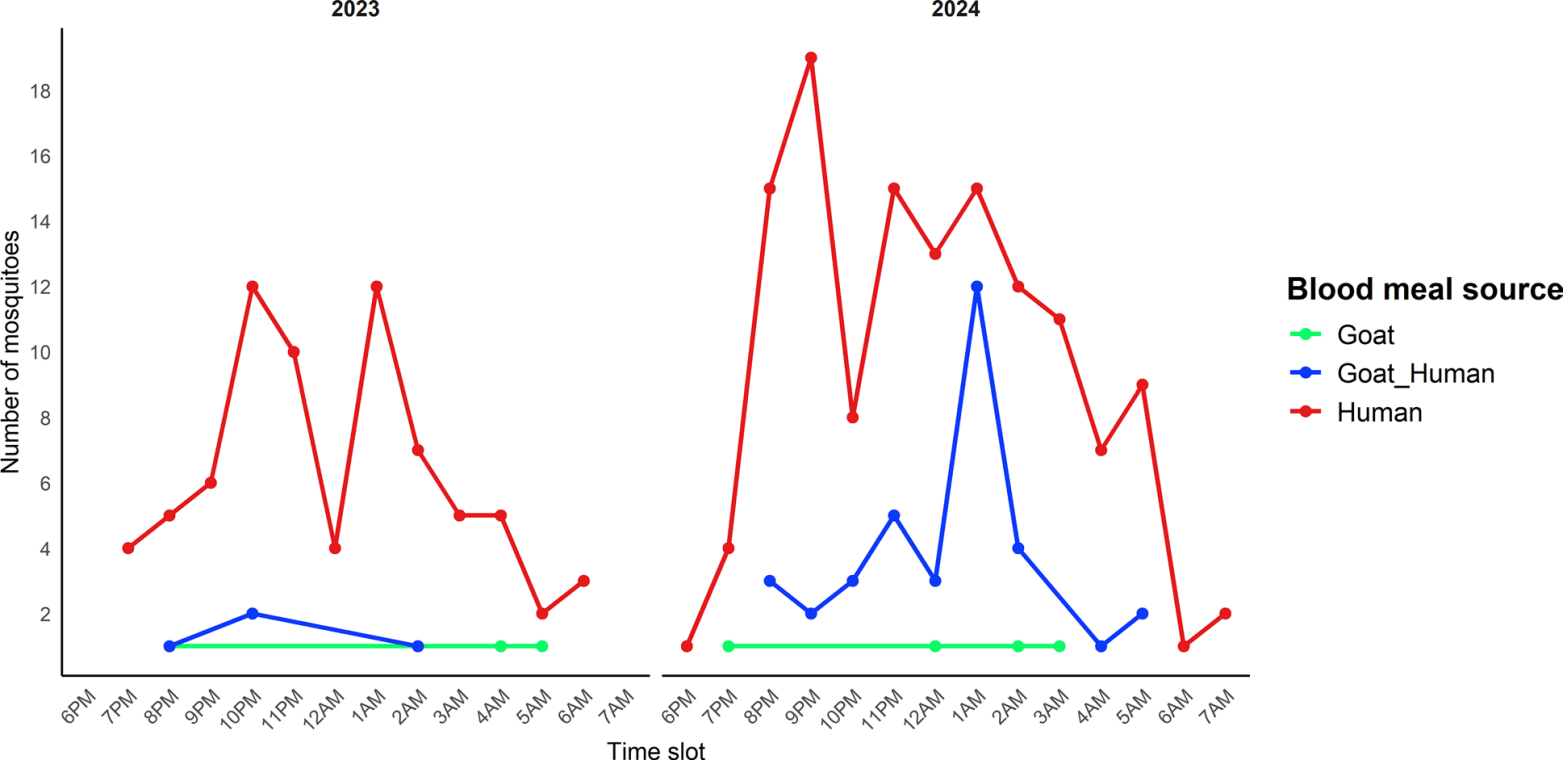
Blood meal sources of *Anopheles* mosquitoes according to biting hours in 2023 and 2024

Temporal profiles of blood-meal sources varied significantly between years (Fig. 7). In both years, humans were the predominant host. In 2024, anthropophagy peaked sharply between 9:00 p.m. and 10:00 p.m., reaching 19 human-fed mosquitoes in a single hour. Mixed human-goat meals were more common in 2024. Exclusive goat blood meals were rare. Biting intensity in 2023 showed two moderate peaks (10:00 p.m and 1:00 a.m.), whereas 2024 exhibited a strong early evening peak followed by sustained activity into early morning.

### *Plasmodium* spp. infection and entomological inoculation rates

All mosquitoes were screened for *P. falciparum*, *P. malariae*, and *P. ovale*. Only *P. falciparum* was detected. Sporozoite-positive samples were found in *An. nili*, *An. coustani*, *An. pharoensis*, and *An. rufipes*. No infections were detected in *An. funestus* in either year (Table 3). Although relatively rare, *An. coustani* had the highest sporozoite rate (30.8%; 4/13). *Anopheles nili* contributed the largest number of infected specimens because of its high abundance. The entomological inoculation rate (EIR) indicated that *An. nili* was the primary driver of transmission:

- EIR 2023: 0.11 infective bites/person/night
- EIR 2024: 0.17 infective bites/person/night

**Table 3:** Malaria transmission drivers of secondary vectors mosquitoes in 2023 and 2024

Years	Species	*n*	*P.f.* positive	HBR	SR	EIR
2023	*Anopheles coustani*	13	4	0.12	0.31	0.04
	*An. funestus*	19	0	0.17	0.00	0.00
	*An. nili*	602	13	5.38	0.02	0.11
	*An. pharoensi*	48	0	0.43	0.00	0.00
	*An. rufipes*	6	1	0.05	0.17	0.01
2024	*An. coustani*	21	1	0.19	0.05	0.01
	*An. funestus*	21	0	0.19	0.00	0.00
	*An. nili*	957	19	8.54	0.02	0.17
	*An. pharoensi*	30	1	0.27	0.03	0.01
	*An. rufipes*	1	0	0.01	0.00	0.00

*An*: *Anopheles*; *n*: number of mosquitoes; *P.f.*: *Plasmodium falciparum*; *HBR*: human binting rate; *SR*: sporozoites rates; *EIR*: entomological inoculation rate

EIR contributions from other species were lower but non-negligible, especially for *An. coustani*.

## Discussion

This study provides a comprehensive assessment of the species composition, spatial and temporal distribution, biting behavior, host-feeding patterns, and *Plasmodium* infection status of secondary malaria vectors across diverse eco-climatic settings in Burkina Faso during 2023–2024. The findings highlight the increasing epidemiological importance of secondary vectors particularly *An. nili* and *An. coustani*, which are often underrepresented in routine surveillance and vector control activities targeting *An. gambiae* s.l.

Consistent with previous studies in West Africa, *An. nili* overwhelmingly dominated the non-*gambiae* s.l. mosquito fauna, accounting for > 87% of all collections. Its predominance in the Sudanian zone, especially in Gaoua, is consistent with its known ecological preference for humid or forest-fringe habitats and its capacity to maintain transmission when primary vector densities decline [34, 35]. The absence of secondary vectors in the Sahelian zone reflects ecological constraints related to aridity and limited larval habitats [36]. The highest species richness observed in Diébougou further underscores the influence of environmental heterogeneity on vector assemblages [37]. Interannual shifts in species composition such as the dominance of *An. pharoensis* in 2023 and *An. coustani* in 2024 in the Sudano-Sahelian zone suggest ecological plasticity and sensitivity to rainfall variability. Positive correlations between rainfall and the abundance of *An. coustani*, *An. nili*, and *An. funestus* align with earlier observations in Burkina Faso and neighboring countries [38, 39]. Conversely, the slight negative association between rainfall and *An. funestus* abundance in multivariate analysis may reflect complex interactions among microhabitats, density dependence, and local vector-control pressures [40].

Biting-behavior analyses demonstrate that *An. nili* maintains high human biting rates (up to 11.9 bites/person/night), reinforcing its role as a major secondary vector. Its predominantly outdoor biting behavior, together with the exophagy of *An. funestus* and the flexible indoor-outdoor patterns of *An. coustani* and *An. pharoensis*, highlights the limitations of interventions focused exclusively on indoor transmission, such as ITNs and IRS [38, 39]. The presence of species exhibiting early evening biting (*An. coustani*) or extended nocturnal activity (*An. nili*) reflects behavioral shifts increasingly reported across Africa in response to high ITNs coverage [41, 42]. These findings support the growing evidence that secondary vectors contribute substantially to residual malaria transmission once primary vectors have been suppressed [11, 43].

Host-feeding analyses revealed considerable heterogeneity across species and sites. *Anopheles nili* displayed strong anthropophilic tendencies, particularly in Gaoua, where more than two-thirds of all blood meals were human-derived, underscoring its high vectorial competence [44]. In contrast, *An. coustani*, *An. rufipes*, and *An. pharoensis* exhibited predominantly zoophilic feeding patterns, which may reduce but not eliminate their contribution to malaria transmission, especially in areas where humans and livestock cohabit closely [24, 45]. The detection of mixed human-animal blood meals across several species suggests opportunistic feeding and potential “bridge vector” roles, which have been increasingly recognized in malaria transmission ecology [46, 47].

The detection of *P. falciparum* sporozoites in four secondary vector species, *An. nili*, *An. coustani*, *An. pharoensis*, and *An. rufipes*, corroborates growing evidence from across Africa indicating that secondary vectors can sustain malaria transmission, particularly in the context of ecological change and intervention pressure [10, 48, 49]. Notably, *An. coustani* exhibited the highest sporozoite rate (30.8%), despite relatively low abundance. This supports reports from other regions suggesting that *An. coustani* may act as a competent secondary vector under certain ecological conditions [50]. The absence of infected *An. funestus* may reflect local ecological dynamics or sampling limitations, as the species is a known vector in parts of Burkina Faso and elsewhere [51]. The entomological inoculation rates measured in this study indicate that *An. nili* remains the principal contributor to malaria transmission among the secondary vectors, with EIR values increasing from 0.11 to 0.17 infective bites/person/night between 2023 and 2024. These results mirror recent observations of persistent secondary-vector-driven transmission despite widespread ITN coverage [49, 51–53]. The study highlights critical gaps in the current vector-control paradigm, which remains heavily focused on *An. gambiae* s.l. and indoor-resting mosquito populations [54, 55].

Given the demonstrated presence of infected, anthropophilic, and predominantly outdoor-biting secondary vectors, there is a pressing need to integrate complementary interventions capable of targeting outdoor transmission. These may include larval source management, environmental sanitation, spatial repellents, endectocides, and outdoor mosquito traps [55, 56]. Additionally, community-based environmental management and educational initiatives tailored to local ecological conditions are essential to address the heterogeneity of transmission dynamics across regions [57, 58]. Sustained, molecularly informed entomological surveillance will be critical to detecting behavioral shifts, monitoring insecticide resistance, and adapting control strategies in real time [59, 60]. Strengthening such surveillance systems is essential for the long-term effectiveness of integrated malaria-control and elimination efforts in Burkina Faso and similar ecological contexts. Future research will focus on molecular identification of species groups and complexes, thereby enabling a more accurate determination of the specific secondary vector species contributing to transmission.

## Conclusions

This study demonstrates that five *Anopheles* species, *An. coustani*, *An. funestus*, *An. pharoensis*, *An. rufipes*, and *An. nili*, contribute to *Plasmodium* transmission in Burkina Faso alongside *An. gambiae* s.l., the primary malaria vector. Among these species, *An. nili* was the most abundant and widespread, while *An. coustani* exhibited the highest sporozoite infection rate despite its relatively low density. All species except *An. funestus* were found infected with *Plasmodium falciparum*, underscoring their potential role in sustaining malaria transmission. The findings highlight the growing epidemiological relevance of secondary vectors and the complexity of malaria transmission dynamics in Burkina Faso. Their predominantly outdoor and sometimes crepuscular biting behaviors, along with anthropophilic tendencies in key species such as *An. nili*, challenge the effectiveness of interventions that primarily target indoor-biting *An. gambiae* s.l. populations. These results emphasize the need to complement existing vector control tools with strategies that can address outdoor and residual transmission. Potential approaches include larval source management, spatial repellents, outdoor trapping technologies, and targeted environmental management. Community engagement and tailored interventions based on local ecological conditions will also be essential. Strengthened molecular and behavioral surveillance is critical to detect changes in vector ecology, feeding behavior, and insecticide resistance. Incorporating secondary vectors into national monitoring frameworks is necessary to guide adaptive and effective malaria control strategies in Burkina Faso.

## Supplementary Information

Additional file 1.

## Data Availability

Data supporting the main conclusions of this study are included in the manuscript.

## Abbreviations

IRS: Indoor residual spraying
ITNs: Insecticide-treated nets
HLC: Human landing catches
PSC: Pyrethrum spray catches
HBR: Human biting rate
SR: Sporozoite rate
EIR: Entomological inoculation rate
GLMs: Generalized linear models
