# Supplementary material for: Beyond Anopheles gambiae sensu lato: exploring the impact of non-dominant Anopheles species on malaria persistence in high-transmission endemic areas of Burkina Faso
Source: Parasit Vectors. 2026 Jan 5;19:68. doi: 10.1186/s13071-025-07210-2 (PMC12870828; doi:10.1186/s13071-025-07210-2)
Supplement: Supplementary file 1 — Additional file 1. [file 13071_2025_7210_MOESM1_ESM.docx]

**Supplementary Table 1** List of Primers for the blood meal source identification

|  |  |  |  |  |
| --- | --- | --- | --- | --- |
| **PCR** | **Species** | **Primers Sequence 5'-3'** | **Amplicon sizes (pb)** | **Reference** |
| 1 | Universal | Fal F: GGTTGTCCTCCAATTCATGTTA | _ | (Kent and Norris, 2005) |
|  | Sheep | Fal R: CTATCCTACTAATCCTCATCCTCATG | 340 |  |
|  | Donkey | Fal R: CTGGTAATCGTCCATCTAC | 460 |  |
|  | Goat | Fal R: CCTAATCTTAGTACTTGTACCCTTCCTC | 150 |  |
| 2 | Universal | Fal F: GGTTGTCCTCCAATTCATGTTA | _ | (Kent and Norris, 2005) |
|  | Human | Fal R: GGCTTACTTCTCTTCATTCTCTCCT | 350 |  |
|  | Pig | Fal R: CCTCGCAGCCGTACATCTC | 500 |  |
|  | Dog | Fal R: GGAATTGTACTATTATTCGCAACCAT | 750 |  |
|  | Cow | Fal R: CATCGGCACAAATTTAGTCG | 600 |  |
|  |  |  |  |  |
